# *Van Gogh* and *Frizzled* Act Redundantly in the *Drosophila* Sensory Organ Precursor Cell to Orient Its Asymmetric Division

**DOI:** 10.1371/journal.pone.0004485

**Published:** 2009-02-13

**Authors:** José-Eduardo Gomes, Maria Corado, François Schweisguth

**Affiliations:** Ecole Normale Supérieure, CNRS UMR8542, Paris, France; Max-Planck-Institut fuer Neurobiologie, Germany

## Abstract

*Drosophila* sensory organ precursor cells (SOPs) divide asymmetrically along the anterior-posterior (a-p) body axis to generate two different daughter cells. Planar Cell Polarity (PCP) regulates the a-p orientation of the SOP division. The localization of the PCP proteins Van Gogh (Vang) and Frizzled (Fz) define anterior and posterior apical membrane domains prior to SOP division. Here, we investigate the relative contributions of Vang, Fz and Dishevelled (Dsh), a membrane-associated protein acting downstream of Fz, in orienting SOP polarity. Genetic and live imaging analyses suggest that Dsh restricts the localization of a centrosome-attracting activity to the anterior cortex and that Vang is a target of Dsh in this process. Using a clone border assay, we provide evidence that the *Vang* and *fz* genes act redundantly in SOPs to orient its polarity axis in response to extrinsic local PCP cues. Additionally, we find that the activity of *Vang* is dispensable for the non-autonomous polarizing activity of *fz*. These observations indicate that both Vang and Fz act as cues for downstream effectors orienting the planar polarity axis of dividing SOPs.

## Introduction

Asymmetric cell division is a fundamental and evolutionarily conserved process for generating cell diversity throughout metazoan development. This process often relies on the unequal segregation of molecules that regulate the fate of the daughter cells (reviewed in [1], [2]). The molecular mechanisms underlying this process can be studied in sensory organ lineages in *Drosophila*. Each external sensory organ of the adult fly stems from single SOPs through a series of stereotyped asymmetric divisions [3]. In the notum, SOPs divide asymmetrically along the a-p axis of the body to generate an anterior pIIb cell and a posterior pIIa cell [4]. The pIIa vs pIIb binary fate decision relies on the unequal segregation of two regulators of Delta/Notch signaling that localize at the anterior cortex of dividing SOPs. The polar localization of these regulators is controlled by the atypical Protein Kinase C (aPKC)- Par6 complex that localizes at the opposite posterior pole [1], [2], [5].

The a-p orientation of the SOP division relies on the planar cell polarization of the single-layered notum epithelium [4], [6], [7], [8]. A small number of evolutionarily conserved proteins act downstream of global polarity cues to locally coordinate the polarization of epithelial cells perpendicular to their apical-basal axis, i.e. within the plane of the tissue (reviewed in [9], [10]). These include the seven-pass transmembrane protein Frizzled (Fz) [11], the DEP domain-containing protein Dishevelled (Dsh) that interacts with Fz and acts downstream of Fz [12], [13], the four-pass transmembrane protein Van Gogh (Vang) (also known as Strabismus) [14], [15] and the atypical cadherin Flamingo (Fmi) [16]. Mutations in the *fz*, *dsh*, *Vang* and *fmi* genes randomize the orientation of the SOP division relative to the body axis [4], [6], [7], [8]. The mechanisms whereby these PCP proteins act to position the aPKC-Par6 complex and orient the mitotic spindle in SOPs are not known.

Asymmetric localization of Fz and Vang at opposite poles at the apical cortex of epithelial cells is an early read-out for PCP [17], [18]. Asymmetric distribution of Fz and Vang further underlies the local coordination of planar polarization by contributing to the cell-cell propagation of polarity (reviewed in [9], [10]). Additionally, asymmetric localization of Fz and Vang provides subcellular cues for the polarization of the cytoskeleton. The mechanisms whereby Fz and Vang act intracellularly to polarize epithelial cells are partly understood in the context of wing epidermal cells [19], [20]. The extent to which similar mechanisms operate in asymmetrically dividing SOPs remains to be determined. Previous studies have shown that Fz localizes at the posterior cortex of SOPs prior to division whereas Vang accumulates at the anterior apical cortex [8]. Additionally, while Fmi localizes at the apical cortex with no sign of asymmetry in SOPs [7], recent studies have suggested that Fmi associate with either Vang or Fz to form distinct complexes at opposite poles of the cell [21], [22]. The asymmetric distribution of Fz and Vang in dividing SOPs therefore suggests that Fz and/or Vang may act locally to regulate the activity of downstream effectors. However, the relative roles of Vang and Fz in positioning the aPKC-Par6 complex and in orienting the mitotic spindle are, however, still elusive. Here, we have studied the relative contributions of Vang and Fz in orienting the SOP polarity axis at mitosis. We have used a live imaging assay to show that Dsh acts in part by inhibiting Vang to restrict the localization of a centrosome-attracting activity. Using clonal analysis, we have shown that both *Vang* and *fz* act redundantly to orient the SOP polarity axis in response to PCP. These observations indicate that both Vang, at the anterior cortex, and Fz, at the posterior cortex, contribute to the a-p orientation of dividing SOPs.

## Materials and Methods

### Flies

**Figure 1:**
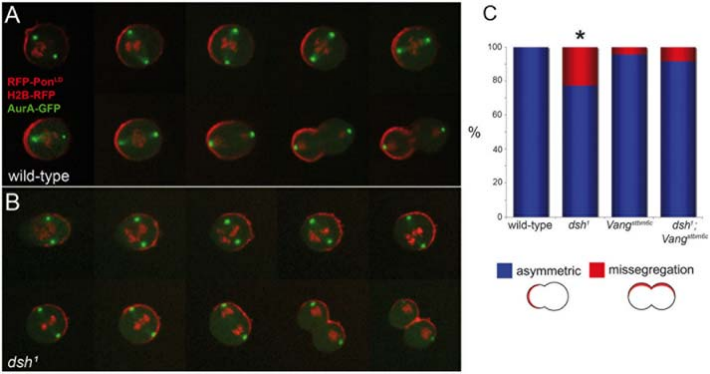
Dsh acts via Vang to inhibit a centrosome-attracting activity at anaphase. (A,B) Time-lapse recording of SOP division in wild-type (A) and *dsh^1^* mutant pupae (B) using RFP-Pon^LD^ (red), Histone2B-RFP (red) AurA-GFP (green). RFP-Pon^LD^ is mis-segregated in the *dsh^1^* mutant SOP. The two centrosomes, marked by AurA-GFP, appear to interact with the cortical domain marked by RFP-Pon^LD^ and moved off-center towards this domain at anaphase. (C) Quantification of the defects in RFP-Pon^LD^ segregation in wild-type, *dsh^1^*, *Vang^stbm6c^* and *dsh^1^ Vang^stbm6c^* double mutant SOPs. The severity of the *dsh^1^* phenotype is significantly different from those associated with all other genotypes in a two-by-two comparison using a Fischer exact test. No other difference in pairwise comparisons was statistically significant.

**Figure 2:**
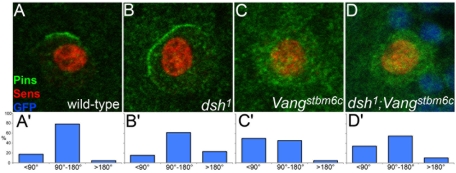
Regulation of Pins cortical localization by Dsh is partly independent of Vang. The cortical localization of Pins (green) was examined in SOPs (Sens, red) in wild-type (A,A′), *dsh^1^* (B,B′), *Vang^stbm6c^* (C,C′) and *dsh^1^ Vang^stbm6c^* double mutant (D,D′) SOPs. In D, nls-GFP (in blue) was used as clonal marker. The extent of the Pins-positive cortical domain in late prophase SOPs was measured as an angle value. The results of this quantification are shown in the bottom panels.

**Figure 3:**
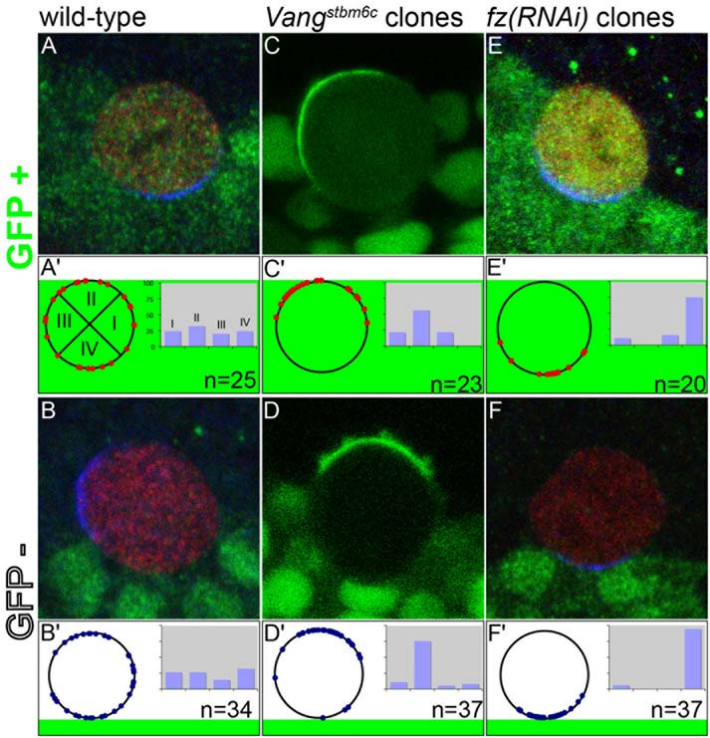
Fz and Vang are individually dispensable to orient the SOP polarity axis. The orientation of SOP division was studied along the border of wild-type control (A–B′), *Vang^stbm6c^* mutant (C–D′) and *fz(RNAi)* (E–F′) clones. SOPs were identified using Sens (red in A, B, E and F) in fixed tissues and GFP-Pon^LD^ (green in C and D) in living tissues. Orientation of the division was determined using Pins (blue in A, B, E and F) or GFP-Pon^LD^ (green in C and D). We used nls-GFP (in green) as a clone marker. (A–B′) wild-type SOPs inside (GFP− in A) and outside (GFP+ in B) control clones. (C–D′) *Vang^stbm6c^* mutant (GFP− in C) and wild-type (GFP+ in D) SOPs. (E–F′) *fz(RNAi)* (GFP− in E) and wild-type (GFP+ in F) SOPs. The orientation of SOP division was measured as an angle between the axis of SOP polarity oriented towards Pins and Pon and a line corresponding to the clone border at the position of the dividing SOPs (see A′ for a graphic representation). Angle values corresponding to the genotypes studied in top panels are plotted in the bottom panels. The orientation of the asymmetry axis, relative to the clone margin, was divided in four categories, corresponding to four 90° quadrants of the circumference (see A′). Statistical differences between genotypes were evaluated by comparing the number of SOPs per quadrant using a Fischer exact test (4×2 contingency table). No statistically significant difference was seen in the orientation of wild-type control SOPs located outside (A′) and inside (B′) the clone. However, the orientation of SOPs located along *Vang^stbm6c^* mutant and *fz(RNAi)* clone borders (C′,D′,E′ and F′) was significantly different from wild-type control distribution.

**Figure 4:**
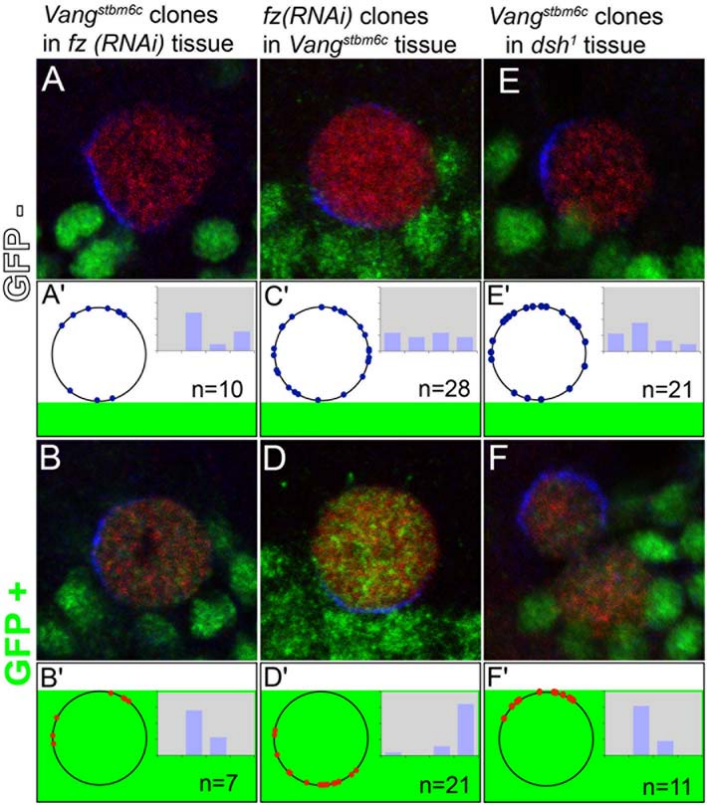
Fz and Vang act redundantly. The orientation of SOP division was studied along the border of *Vang^stbm6c^* mutant clones generated in *fz(RNAi)* pupae (A–B′), of *fz(RNAi)* clones generated in *Vang^stbm6c^* mutant pupae (C–D′) and of *Vang^stbm6c^* mutant clones generated in *dsh^1^* mutant pupae (E–F′). SOPs were identified using Sens (red) and orientation of the division was determined using Pins (blue). Clone borders were marked by nls-GFP (green). (A) *fz(RNAi) Vang^stbm6c^* SOP (GFP−) in *Vang^stbm6c^* mutant pupae. (B) *Vang^stbm6c^* mutant SOP (GFP+) at the border of *fz(RNAi) Vang^stbm6c^* mutant cells. (C) *dsh^1^ Vang^stbm6c^* SOP (GFP−) in *dsh^1^* mutant pupae. (D) *dsh^1^* mutant SOP (GFP+) at the border of *dsh^1^ Vang^stbm6c^* mutant cells. (E) *fz(RNAi) Vang^stbm6c^* SOP (GFP−) in *fz(RNAi* mutant pupae. (D) *fz(RNAi)* SOP (GFP+) at the border of *fz(RNAi) Vang^stbm6c^* mutant cells. Angle values corresponding to the genotypes studied in top panels are plotted in bottom panels as in Figure 3. Statistical differences were evaluated using Fisher exact test (4×2 contingency table). In all three genotypes, the orientation of SOPs located inside the clone (A′, C′ and E′) was not statistically different from the random distribution seen in wild-type control clones (see Fig. 3A′,B′). In contrast, the orientation of SOPs located outside the clone (B′, D′ and F′) was significantly different from the random distribution seen in wild-type control clones (see Fig. 3A′,B′). Additionally, no statistically significant differences were observed in the orientation of SOPs located either outside *Vang^stbm6c^* mutant clones in *fz(RNAi)* pupae (B′) or *dsh^1^* mutant pupae (F′) and wild-type pupae (Fig. 3C′). Similarly, no statistically significant difference was observed in the orientation of SOPs located outside *fz(RNAi)* clones in *Vang^stbm6c^* mutant (D′) and wild-type pupae (Fig. 3E′).

The following genotypes were studied:

Figure 1:
  1. *neurA-*Histone2B-RFP/+; UAS-RFP-Pon^LD^/+; neurP72GAL4 UAS-AurA-GFP/+
  2. *neurA-*Histone2B-RFP/+; UAS-RFP-Pon^LD^ *Vang^stbm6c^*/*Vang^stbm6c^*; neurP72GAL4 UAS-AurA-GFP/+
  3. *Ubx-flp*/*neurA-*Histone2B-RFP; UAS- RFP-Pon^LD^ *Vang^stbm6c^* FRT42D/*ubi-nlsGFP* FRT42D; neurP72GAL4 UAS-AurA-GFP/+;
  4. *dsh^1^*/Y; UAS-RFP-Pon^LD^/+; neurP72GAL4 UAS-AurA-GFP/*neurA-*Histone2B-RFP
  5. *Ubx-flp dsh^1^*/Y; UAS-RFP-Pon^LD^ *Vang^stbm6c^* FRT42D/*ubi-nlsGFP* FRT42D; neurP72GAL4 UAS-AurA-GFP/*neurA-*Histone2B-RFP
Figure 2:
  6. *Ubx-flp*/+; *Vang^stbm6c^* FRT42D/*ubi-nlsGFP* FRT 42D
  7. *dsh^1^*/Y
  8. *Ubx-flp dsh^1^*/Y; *Vang^stbm6c^* FRT42D/*ubi-nlsGFP* FRT42D
Figure 3:
  9. *hs-flp/+*; ; *Tubα1>GFP,y^+^>Gal4*/+
  10. *Ubx-flp*/+; *Vang^stbm6c^* FRT42D/*ubi-nlsGFP* FRT42D; *neur*P72GAL4/UAS-GFP-Pon^LD^
  11. *hs-flp/+*; ; UAS-*fz(RNAi)*/*Tubα1>GFP,y^+^>Gal4*
Figure 4:
  12. *Ubx-flp dsh^1^*/Y; *Vang^stbm6c^* FRT42D/*ubi-nlsGFP* FRT42D
  13. *Ubx-flp*/+; *Vang^stbm6c^* FRT42D/*ubi-nlsGFP* FRT42D; UAS-*fz(RNAi)*/*tub*-*Gal4*
  14. *hs-flp*; *Vang^stbm6c^*; UAS-*fz(RNAi)*/*Tubα1>GFP*,*y^+^>Gal4*

The following alleles and constructs were used: *Vang^stbm6c^* ) [15], *dsh^1^* [12], UAS-fz(RNAi) [23], Ubx-flp (from J. Knoblich), *Tubα1>GFP,y^+^>Gal4* [24], *neur*P72GAL4 [6], UAS-Histone2B-RFP [25], UAS-GFP-Pon^LD^ [26], UAS-AurA-GFP [27] and UAS-RFP-Pon^LD^ [28]. The *neur*-Histone2B-RFP transgene encodes the Histone2B::RFP fusion protein [25] under the control of *neur* cis-regulatory sequences that drive SOP-specific expression (E. Lai, personal communication). Transgenic flies were generated by P-element transformation.

### Immunohistochemistry and imaging

Live imaging was carried out as described [6]. Pupal nota were dissected and processed as previously described [3]. Primary antibodies and dilutions were: guinea pig anti-Senseless (Sens; 1∶3000; from H. Bellen); rat anti-Pins (1∶1000; from P. Bryant) and rabbit anti-GFP (Molecular Probes; 1∶1000). Cy2, Cy3- and Cy5-coupled secondary antibodies were from Jackson's Laboratories

Orientation of SOP division was either measured at telophase in living pupae using GFP-Pon^LD^ or on fixed tissue using Pins as a polarity marker. Quantification of the fate determinants mis-segregation phenotype was performed in living pupae using RFP-Pon^LD^ as a marker for the localization of fate determinant.

All images were acquired on a Leica SP2 confocal microscope and assembled using NIH ImageJ and Photoshop softwares. Angles were measured using ImageJ.

## Results

### Dsh restricts the extent of a centrosome-attracting activity

Wild-type SOPs divide along the a-p axis. In contrast, the orientation of SOP division is random relative to the a-p axis in all PCP mutants studied so far. Additionally, previous studies have shown that loss of *dsh* or *fz* PCP activity not only randomizes the orientation of dividing SOPs but also results in defects in the unequal segregation of Numb and Partner of Numb (Pon) axis [4], [6], [8]. In wild-type SOPs, Pon localizes in a crescent at the anterior cortex during prophase and prometaphase and segregates into the anterior cell at anaphase because the cell division plane is perpendicular to the crescent. In most *fz* or *dsh^1^* mutant SOPs, the cell division plane is perpendicular to the crescent, thereby leading to normal segregation of Numb and Pon. However, in 11–22% of the *fz* and *dsh^1^* mutant SOPs, the cortical domain where Pon accumulates is bisected at anaphase [6], [8] (Fig. 1). This correlates with a misalignment of the mitotic spindle with the Pon crescent [6]. This was interpreted to suggest that both poles of the mitotic spindle, instead of a single one, interact with the cortical Pon domain in *fz* and *dsh^1^* mutant SOPs [8]. This interpretation implied that an activity attracting the centrosomes colocalizes with Pon at the cortex and that the role of PCP factors is to localize and restrict this activity to the anterior pole of the cell.

To directly test whether the two centrosomes actually move towards the Pon domain in *dsh^1^* mutant SOPs, we have used live imaging to monitor centrosome dynamics using the centrosomal marker AurA-GFP (AuroraA fused to the Green Fluorescent Protein). AurA-GFP was specifically expressed in SOPs together with RFP-Pon^LD^ (Red Fluorescent Protein fused to the localization domain (LD) of Pon and an Histone2B-RFP marker. We first confirmed that 22% (n = 40) of the *dsh^1^* mutant SOPs mis-segregate RFP-Pon^LD^ (Fig. 1; wild-type control: 0%, n = 36). Additionally, we observed that both centrosomes localize close to the RFP-Pon^LD^ domain at metaphase in the *dsh^1^* mutant SOPs that mis-segregate RFP-Pon^LD^, and that both centrosomes moved off-center towards the Pon domain at anaphase (Fig. 1B). This suggests that a centrosome-attracting activity localizes in the Pon domain. In contrast, a single centrosome appears to be associated at metaphase with the cortical domain containing RFP-Pon^LD^ in the remaining 78% of the *dsh^1^* mutant SOPs that unequally segregate RFP-Pon^LD^, as wild-type SOPs do. These data therefore suggest that loss of *dsh* PCP activity not only result in the randomization of the SOP division axis but also in an extension of a cortical activity that pulls on the centrosomes. This cortical domain would be too small in wild-type SOPs to allow for interaction with the two centrosomes, but would be large enough in *dsh^1^* mutant SOPs for both centrosomes to interact with it, thereby leading to defects in the segregation of RFP-Pon^LD^ at anaphase. These data therefore suggest a model whereby Dsh acts at the posterior cortex, downstream of Fz, to restrict the localization of a centrosome-attracting activity to the anterior cortex. This centrosome-attracting activity likely involves the recently identified Gαi-Pins-Mud complex that localizes at the anterior cortex and regulates centrosome-cortex interaction [29], [30], [31], [32]. Consistent with this interpretation, a loss in *dsh* PCP activity leads to an increased accumulation of Pins at the cortex in prophase [8] (see also Fig. 2).

### Dsh acts in part via Vang

In contrast with *dsh*, loss of *Vang* activity had no major effect on Pon segregation (Fig. 1C) [8]. Additionally, loss of Vang resulted in a delay in the recruitment of Pins at the cortex, indicating that Vang plays a positive role in recruiting Pins. Moreover, overexpression of Vang, together with its partner Prickle, led to mis-segregation of Pon and increased Pins recruitment [8]. Since the cortical localization of Vang is no longer restricted to one pole in *dsh* mutant SOPs [8], we hypothesize that mislocalization of Vang in *dsh^1^* mutant SOPs may account for the extended localization of the centrosome-attracting activity. Accordingly, Dsh would act upstream of Vang by inhibiting the cortical localization of Vang which would in turn positively regulate the localization of the proposed centrosome-attracting activity. Alternatively, Vang may act upstream of Dsh, with Dsh restricting the extent of the proposed centrosome-attracting activity in a manner that does not involve Vang. To distinguish between these two models, we have examined whether the segregation defect seen in *dsh^1^* mutant SOPs depends on the presence of Vang. To do so, the segregation of RFP-Pon^LD^ was examined in *dsh^1^ Vang* double mutant SOPs. Mis-segregation of RFP-Pon^LD^ was seen in 8% of the *dsh^1^ Vang* double mutant SOPs (n = 97; Fig. 1C). The frequency of this defect is not statistically different from the one observed in *Vang* mutant cells (4% of mis-segregation; n = 212) but appears to be statistically different from the one measured in *dsh^1^* mutant pupae (22%; n = 40; Fig. 1C). Thus, *Vang* appears to be epistatic to *dsh^1^* for the RFP-Pon^LD^ mis-segregation phenotype, suggesting that Vang acts, at least in part, downstream of Dsh to promote centrosome-cortex interaction and orient the spindle.

To further examine epistasis between *dsh^1^* and *Vang*, we analyzed the localization of Pins in *dsh^1^ Vang* double mutant SOPs. In wild-type SOPs, Pins localizes at the anterior apical cortex starting at late prophase prior to nuclear envelope breakdown (Fig. 2A) [8]. Quantification of the size of the Pins crescent indicated that the cortical domain marked with Pins extends over 25–50% (90–180° in Fig. 2A′) of the anterior cortex in most wild-type SOPs (78%, n = 23). We further confirmed that Vang promotes the cortical localization of Pins: 50% of the *Vang* mutant SOPs (n = 22) showed either no crescent or a crescent smaller than 25% of the cortex (i.e. <90°; Fig 2C,C′) [8]. We also confirmed that Dsh antagonizes the recruitment of Pins at the cortex: 23% of the *dsh^1^* mutant SOPs (n = 26) had a crescent covering at least 50% of the cortex (Fig. 2B,B′) [8]. The phenotype of *dsh^1^ Vang* double mutant SOPs appeared to be intermediate between the *Vang* and the *dsh^1^* phenotype, with 34% of the *dsh^1^ Vang* mutant SOPs (n = 29) showing either no crescent or a crescent smaller than 25% of the cortex (Fig. 2D,D′) and 10% of the double mutant SOPs having a crescent covering at least 50% of the cortex. Taken together, our data suggest that Dsh acts only in part via Vang and that the activities of both Vang and Dsh may contribute to spindle orientation in dividing SOPs.

### Orientation of the planar polarity axis in response to PCP does not depend on *Vang* and *fz* activities in SOPs

In order to examine the relative contributions of Vang and Fz/Dsh in orienting polarity in dividing SOPs, we have created an experimental situation in which SOPs are predicted to have asymmetric Fz in the absence of Vang, or asymmetric Vang in the absence of Fz. To do so, we have taken advantage of the non-autonomous activity of the *Vang* and *fz* genes [14], [33]. Previous studies have established that *Vang* and *fz* mutations have a non-autonomous effect in the wing epithelium such that wild-type epithelial cells in contact with mutant cells orient their polarity relative to the clone border and not relative to the body axis (see [19] for a detailed analysis). We first verified that *Vang* and *fz* act non-autonomously in the developing notum by examining the orientation of dividing wild-type SOPs that are in contact with either *Vang* mutant cells or *fz(RNAi)* cells with strongly decreased *fz* activity. The orientation of dividing SOPs was monitored using either GFP-Pon^LD^ or Pins. In these experiments, the position of the mitotic spindle was inferred from the orientation of dividing cells at anaphase. We found that wild-type SOPs orient towards *Vang* mutant cells, with GFP-Pon^LD^ accumulating at the contact region between wild-type and *Vang* mutant cells (Fig. 3C,C′). Conversely, wild-type SOPs orient away from *fz(RNAi)* cells, with Pins accumulating opposite to the contact region with cells that have low *fz* activity (Fig 3E,E′). In control wild-type clones, SOPs divide with a stereotyped orientation relative to the a-p body axis (not shown) but randomly relative to the clone border (Fig. 3A–B′), indicating that the position of the clone border is unbiased relative to the a-p orientation of the tissue. Thus, any bias observed in SOP orientation along mutant clones should result from the mutant genotype. We therefore conclude that both *Vang* and *fz* act non-autonomously to influence the polarity of wild-type SOPs in the developing notum. This conclusion is entirely consistent with studies in wing epithelium where groups of *Vang* and *fz* mutant cells have a domineering non-autonomous effect and cause neighboring wild-type cells to mispolarize relative to the main proximal-distal axis.

We then examined the orientation of dividing *Vang* mutant SOPs that are in direct contact with wild-type cells. We observed that, in these cells, GFP-Pon^LD^ localizes away from the clone border (Fig. 3D,D′). We interpret this observation based on the local coordination of cell polarity such that, in *Vang* mutant SOPs, Fz preferentially localizes at the contact region with wild-type cells. Thus, localized Fz activity appears to regulate in a cell-autonomous and *Vang*-independent manner the localization of GFP-Pon^LD^ at the opposite pole. We therefore conclude that *Vang* mutant SOPs can properly respond to PCP cues generated at the clone border. This is consistent with the notion that Vang is not the only activity that can orient the SOP polarity axis in response to local PCP information.

We reciprocally analyzed the orientation of the *fz(RNAi)* dividing SOPs that are located along the clone border. Pins was found to localize towards the clone border. Thus, SOPs with reduced levels of *fz* activity can properly orient their polarity axis in response to PCP signals generated at the clone border. We interpret this observation to suggest that Vang preferentially localizes at the contact region with wild-type cells and regulates, in a *fz*-independent manner, the localization of Pins at this pole. Together, these data indicate that the activities of Vang and Fz are not, on their own, essential in SOPs to orient their axis of polarity in response to local PCP cues generated along the clone border.

### *Vang* appears to act redundantly with *fz* and *dsh* in the SOP to orient its polarity axis in response to extrinsic PCP cues

One interpretation of our results is that Fz and Vang can act independently of one another in SOPs to orient asymmetric division in response to extrinsic PCP cues. Accordingly, the activities of *fz* and *Vang* would act in a redundant manner in SOPs to orient its planar polarity. To test this hypothesis, we have analyzed the orientation of the polarity axis in *Vang fz(RNAi)* SOPs located at the border of *Vang* mutant or *fz(RNAi)* clones. To do so, we generated clones of *fz(RNAi)* cells in *Vang* mutant pupae as well as *Vang* mutant clones in *fz(RNAi)* pupae. In both cases, we found that the polarity axis of *fz(RNAi) Vang* SOPs is randomly oriented relative to the clone border (Fig. 4A,A′ and C,C′). We can exclude that this defective orientation results from a defect in the generation of planar polarity cues at the clone border since both *fz(RNAi)* (Fig. 4B,B′) and *Vang* mutant SOPs (Fig. 4D,D′) appear to orient their polarity axis relative to the border of the *Vang* and *fz(RNAi)* clones, respectively. Similarly, the polarity axis of *dsh^1^ Vang* double mutant SOPs is randomly oriented relative to the clone border (Fig. 4E,E′), whereas *dsh^1^* mutant SOPs respond to PCP cues from the *Vang* clone border to orient their polarity axis (Fig. 4F,F′). These data strongly suggest that the *Vang* gene acts redundantly with the *fz* and *dsh* genes to orient the SOP polarity axis in response to extrinsic PCP cues generated at clone borders. We therefore propose that planar orientation of dividing SOPs in response to local polarity cues is regulated by both ‘anterior’ cues via the localized activity of Vang and by ‘posterior’ cues via the localized activity of Fz/Dsh.

Additionally, our observation that *fz^+^* SOPs orient their polarity axis relative to the position of the *fz(RNAi)* cells in the absence of *Vang* activity (Fig. 4D,D′) argues that the non-autonomous activity of *fz* is independent of the activity of the *Vang* gene. Conversely, *Vang^+^* SOPs can orient their polarity axis relative to the position of the *Vang* mutant tissue despite the strong loss of *fz* (Fig. 4B,B′) and *dsh* (Fig. 4F,F′) PCP activities in *fz(RNAi)* and *dsh1* pupae, respectively. This observation raises the possibility that the non-autonomous activity of *Vang* may be independent of the PCP activities of *fz* and *dsh*.

## Discussion

Prior to this study, the role of PCP genes in orienting asymmetric SOP division had only been studied in single mutant pupae [4], [6], [7], [8]. While these studies have clearly established a role for PCP genes in orienting asymmetric cell divisions in the notum, the relative roles of the anterior and posterior PCP complexes in orienting the polarity axis were not addressed. Here, we have used clonal analysis and double mutant combinations to investigate the relative contributions of Vang, a key component of the anterior complex, and Fz/Dsh, two components of the posterior complex. We find that Vang and Fz act redundantly to orient the SOP planar polarity axis, with Dsh acting only in part by antagonizing the cortical localization of Vang. We also find that cells lacking both Fz and Vang can influence the planar orientation of neighboring SOPs.

Our data on SOP orientation along clone borders indicates that *Vang* mutant and *fz(RNAi)* SOPs orient their polarity axis relative to the position of their wild-type neighbors. Thus, planar polarization of SOPs is influenced by local cell-cell communication regulated by Vang and Fz. Moreover, Vang and Fz do not play an essential cell-autonomous role to orient the mitotic spindle and specify the position of the ‘anterior’ domain that recruits Pon. Planar polarization along clone border can be interpreted on the basis on the known localization of Fz in *Vang* mutant cells (and of Vang in *fz* mutant cells) at the cortical edges that are in direct contact with wild-type cells [17], [18], [19]. In *Vang* mutant cells, Fz accumulation at contact regions with wild-type cells provides a ‘posterior’ cue that is sufficient to orient the division of *Vang* mutant SOPs. Conversely, in *fz(RNAi)* cells, Vang accumulation towards wild-type cells provides an ‘anterior’ cue that is sufficient to orient the division of *fz(RNAi)* mutant SOPs. Accordingly, both ‘anterior’ cues and ‘posterior’ cues appear to regulate the planar orientation of the SOP division. However, loss of both polarity cues in *Vang dsh* and *Vang fz* double mutant SOPs results in random orientation relative to the clone border. Thus, we propose that posterior and anterior PCP complexes act redundantly within SOPs to orient its planar polarity. This conclusion is in agreement with the one reached by Strutt and Warrington (2008) for the regulation of trichome position within each wing epidermal cells. Using a similar clone border assay, these authors demonstrated that the site of prehair initiation that prefigures the position of trichome is controlled by an inhibitory ‘proximal’ cue and a positively-acting ‘distal’ cue and that the localized activity of either Fz or Vang is sufficient to specify the site of prehair initiation relative to the clone border [19].

Consistent with the notion that these two PCP complexes act redundantly, we interpret the lack of clear epistatic relationship between the *Vang* and *dsh* genes for the localization of Pins at the cortex to suggest that there is no strict linear relationship between the activities of the posterior and anterior PCP complexes. Thus, distinct anterior and posterior effectors may act downstream of Fz/Dsh and Vang/Pk to determine the position of the Pins/Mud and aPKC/Par6 cortical domains and to orient the spindle. Based on our live-imaging analysis of *dsh* and *dsh Vang* mutant SOPs, we postulate the existence of at least one effector acting downstream of Vang that regulates spindle orientation. Indeed, our analysis supports the notion that a Vang-dependent activity colocalizing with Pon pulls on centrosomes at anaphase. A good candidate for this activity is the Pins/Mud complex that is recruited at the anterior cortex in a manner that depends, at least in part, on the activity of Vang [8]. Additional effectors of Vang and Fz still need to be identified to account for the redundant activities of Fz and Vang. Of note, a limited numbers of effectors acting downstream of Fz and Vang in wing epithelial cells have been shown to specify the cortical site of trichome formation, in part by regulating actin dynamics [19], [20], [34]. Whether these effectors also participate in the planar polarization of SOPs remain to be investigated.

Finally, we found that *fz(RNAi) Vang* cells, that are homozygous for a null allele of *Vang* and have strongly reduced *fz* activity, can modulate the polarity of neighboring SOPs that are either *fz^+^ Vang* or *fz(RNAi) Vang^+^*. Our observation that *Vang fz+* SOPs orient their polarity axis relative to the border of *fz(RNAi)* clone generated in *Vang* pupae is consistent with the observation that Fmi accumulates in a polarized manner along the border of *fz* clones generated in *Vang* mutant wings [22]. Thus, the activity of Vang appears to be dispensable for the non-autonomous activity of Fz in both wing epidermal cells and notal SOPs. We also observed that *Vang^+^ fz(RNAi)* SOPs orient their polarity axis relative to the border of *Vang* clone generated in *fz(RNAi)* pupae. One interpretation is that the activity of Fz is dispensable for the non-autonomous activity of Vang. Accordingly, Vang proteins in *Vang^+^* SOP might localize at the cortical region abutting *Vang* mutant cells even in the absence of Fz. However, this interpretation does not easily fit with the observation that Fmi fails to accumulate in a polarized manner along the border of *Vang* clones generated in *fz* mutant wings [22]. It may thus be that asymmetric distribution of Vang is differently regulated in SOPs and in wing epidermal cells. Alternatively, it is possible that low levels of *fz* activity persists in *fz(RNAi)* pupae and that the non-autonomous polarizing activity of *Vang* seen in our assay depends on this residual activity.

In summary, this study indicates that orientation of the planar polarity axis of dividing SOPs more likely emerges from a network of molecular activities downstream of both Vang and Fz rather than from a linear signaling pathway downstream of spatially-localized Fz. Additionally, our clone border analysis of double mutant combination provides a precise and quantitative approach to further dissect the molecular mechanisms acting downstream of either Vang or Fz that are involved in orienting the SOP asymmetric division.
